# The Mass Loss and Humification of Stumps and Roots in Masson Pine Plantations Based on Log File Records

**DOI:** 10.1371/journal.pone.0160913

**Published:** 2016-08-11

**Authors:** Jiao Zhou, Fuzhong Wu, Wanqin Yang, Bo Tan, Zhenfeng Xu, Jian Zhang, Fei Duan, Hui Liu, Meta Francis Justine

**Affiliations:** 1 Long-term Research Station of Alpine Forest Ecosystems, Institute of Ecology & Forestry, Sichuan Agricultural University, Chengdu, 611130, China; 2 Collaborative Innovation Center of Ecological Security in the Upper Reaches of Yangtze River, Chengdu, 611130, China; Pacific Northwest National Laboratory, UNITED STATES

## Abstract

Stumps account for a large proportion of coarse woody debris in managed forests, but their decay dynamics are poorly understood. The loss of mass and the degree of humification of the above-ground woody debris, below-ground woody debris, bark and root system (R1, 10 mm ≥ diameter > 0 mm; R2, 25 mm ≥ diameter >10 mm; 100 mm ≥ R3 > 25 mm; R4 > 100 mm) of Masson pine (*Pinus massoniana*) stump systems were evaluated in southwestern China in a chronosequence of plantations cut 1–15 years prior to the study. The results indicated that above-ground woody debris decomposed more quickly than below-ground woody debris and bark, whereas the degree of humification followed the opposite trend. Compared with one-year stumps, the mass losses of 15-year stump systems were 60.4% for above-ground woody debris, 42.1% for below-ground woody debris, 47.3% for bark, 69.9% for R1, 47.3% for R2, 51.0% for R3, and 83.2% for R4. In contrast, below-ground woody debris showed a greater degree of humification compared with other components in the stump system. Among the root system, fine roots (R1, diameter ≤ 10 mm) had the largest *k* value (0.09), whereas the decay rate of coarser roots (R2, R3, R4; diameter > 10 mm) increased with increasing root diameter. However, coarse roots showed a larger degree of humification (0.2–0.6) than fine roots (0.3–0.4). These results suggest that below-ground woody debris and coarse roots may display a higher degree of humification, showing greater short-term contributions to overall humification when compared with the other components in the stump system.

## Introduction

Stumps consist of above- and below-ground coarse woody debris and roots with different diameters, which are equivalent to approximately 20% of the total living tree biomass in most plantations [1, 2]. The stumps retained in a plantation after logging play important roles in maintaining soil fertility, sequestering carbon, and nursing biodiversity [3–6]. Therefore, understanding of stumps and the root decay process in plantations can provide a scientific basis for sustainable plantation management. Humification is the biogeochemical transformation of woody debris into more recalcitrant humic substances, which is closely related to soil organic carbon sequestration, soil aggregate formation and nutrient stocks [7]. On most plantations, more than 20% of stump biomass is stored underground [8], suggesting that the humification of stumps plays essential roles in soil development [9]. However, the decomposition and humification of stumps and their components are poorly understood.

Theoretically, there are two interdependent processes during the decay of stumps: mass loss and humification. Our understanding of the stump decomposition process remains insufficient for the following reasons. First, stumps are the main coarse woody debris on plantations, wood decomposition occurs over an extremely long time scales due to the low decay rate [10], and complete wood decay under certain conditions can take centuries [11]. Therefore, direct methods for describing the dynamics and rates of woody debris decay are difficult [12], which makes the results of previous studies inaccurate. Second, integrated stump systems include different components that have different diameters, and this problem is often ignored in studies. Moreover, the stump quality is not homogeneous, meaning that studies focused on only part of a stump system may ignore important decay mechanisms that are important to the system as a whole [13]. To obtain information on the roles of stump decomposition in sequestering carbon and the nutrient supply in plantations, previous studies that focused on the decay of stumps and roots mainly studied the nutrient cycling and stocks [14–19], with little attention to humification dynamics, especially simultaneous measurements of the decay of different components of stump systems. Last but not least, stumps often include above- and below-ground parts, both bark and roots with different diameters. Additionally, the site conditions form an important controlling factor of coarse woody debris (CWD) decomposition [20], and each part of the stump system has a different external environment and different decomposer communities, which might influence the rate of stump decay. In managed plantations, annual logging tends to leave numerous stumps on plantations, which might produce a series of decomposing stumps. Therefore, a chronosequence method based on log file records might be an efficient way to understand the short-term process of stump decomposition.

*Pinus massoniana* (Masson pine or Chinese red pine) is a major afforestation tree species in southern China and the Yangtze River Basin. Currently, Masson pine plantations cover approximately 1.43 million hectares in China and play important roles in supplying wood, sequestering carbon, and conserving soil [21]. In the area planted with Masson pine, continuously decaying stumps are retained after annual logging. These decaying and decayed stumps play essential roles in maintaining site fertility, nursing soil biodiversity, and sequestering carbon. Moreover, these continuously decaying stumps provide materials for studying the decaying process of Masson pine stumps. There is a lack of information regarding the decay rate and humification of Masson pine stumps and their linked roots. Therefore, a chronosequence (1–15 years) of Masson pine stump based on log file records was used to study the decay process of Masson pine stumps and linked roots in Masson pine plantations. The objectives of this study are (1) to obtain information on the mass loss and humification processes across a chronosequence of decaying Masson pine stump system and (2) to understand the changes in decay rate and humification based on above- and below-ground woody debris and root diameters in Masson pine plantations. The results are expected to provide a scientific basis for sustainable management of Masson pine plantations.

## Materials and Methods

### Ethics statement

We received permission from the Sichuan Forestry Bureau to conduct scientific experiments in Yibin Laifu Forestry Management Institute in 2013. The mass loss and humification of the stumps and root system evaluated in this study were only sampled at a very limited scale, and our work thus had negligible effects on the function of the broader ecosystem. Moreover, this research was carried out in compliance with the laws of the People’s Republic of China. The research did not involve measurements of humans or animals, and no endangered or protected plant species were involved.

### Study sites and experimental design

This study was conducted in a Masson pine (*P*. *massoniana*) plantation area under the administration of the Laifu Forestry Management Institute in the southeast of China, which was formed in 1956. The study site is located in the upper reaches of the Yangtze River and Sichuan Basin, and it belongs to Gao County, Sichuan Province, China, and is 453 m above sea level (28°34′–28°36′N, 104°32′–104°34′E). The area has a subtropical humid monsoon climate with an annual total mean rainfall of 1021 mm. The annual mean temperature is 18.1°C; the lowest and highest mean temperatures are 7.8°C (in January) and 36.8°C (in July). The soil type in the study area is classified as acidic Alfisol. To supply commercial wood, extensive forestland has been transformed into Masson pine plantations, comprising the tree layer, the shrub layer and the herb layer. The dominant overstory vegetation in all stand ages is *P*. *massoniana*. The shrub layer includes *Rubus pirifolius*, *Viburnum setigerum*, and *Myrsine africana*; the herb layer includes grasses, such as *Pteridium aquilinum*, *Dicranopteris dichotoma*, and *Setaria plicata*. Continuous logging and planting by the institute has left continuously decaying stumps in the age-sequential plantations, providing important experimental materials for investigating the decay process of both stumps and the related roots.

In August 2013, based on log file records from the Laifu Forestry Management Institute, we measured the mass loss and humification of Masson pine stumps and the related roots retained in the plantations after logging from 1999 to 2013. To obtain relatively consistent materials with respect to the initial quality, only the stumps from 29- to 30-year-old trees were investigated and measured in this study. The investigated stumps were obtained from similar forest stands, i.e., having the same soil type and both a similar slope gradient and a similar slope direction. At all stands, the Masson pine plantations were replanted after logging.

### Sampling and analyses

A chronosequence (1–15 years) of decaying stumps was constructed. However, a lack of decaying stump systems was identified for a 6-year period because the rotation sites in 2008 differed from those in other years. After establishing 14 experimental plantations, three 20 m × 20 m (400 m^2^) blocks were established at each plantation. Accordingly, a total of 42 sampling blocks measuring 20 m × 20 m in size, with a distance of 500 m between neighbors, were established. The diameter of all stumps in each forest stand was recorded. Moreover, we sampled soil at 0–30 cm because the roots are mainly distributed in the 0–30 cm soil layer. Three samples were taken in each sample plantation, and a total of 42 (14 × 3 = 42) soil samples were taken. The basic characteristics of the sample plantation are shown in Table 1. We chose one stump in each sampling block and entirely excavated the stump system. To obtain more detailed information on the decay process of complete stump systems, we ensured the integrity of both the above- and below-ground components of the stump systems during the excavation process. The stump systems were classified as above-ground coarse woody debris (diameter ≥ 10 cm and height < 1 m), below-ground coarse woody debris (diameter ≥ 15 cm and height < 1 m), bark and root system.

**Table 1:** The basic characteristics of the sample plantation.

Years of decay of sample plantation	Aspect/Slope (°)	Number of stumps	Stump diameter (cm)	Soil pH	Soil bulk density (0–30 cm) (g·cm^–3^)	Soil organic matter (g·kg^-1^)
1	NW/21	2050	16.4±0.5	4.2	1.4	29.9
2	NW/18	2315	15.9±0.7	4.1	1.4	29.0
3	NW/20	2100	17.2±0.5	4.2	1.5	27.7
4	NW/21	2070	16.6±0.4	4.1	1.4	27.4
5	NW/17	2405	15.6±0.6	4.2	1.4	28.4
7	NW/20	2150	16.2±0.4	4.2	1.4	27.4
8	NW/19	2190	16.4±0.4	4.2	1.4	27.5
9	NW/20	2130	16.3±0.5	4.2	1.5	27.6
10	NW/18	2350	15.9±0.6	4.2	1.4	28.2
11	NW/19	2200	16.1±0.3	4.2	1.4	28.1
12	NW/20	2085	17.1±0.5	4.2	1.4	28.6
13	NW/21	2070	16.7±0.3	4.2	1.4	29.3
14	NW/20	2125	16.8±0.5	4.2	1.4	30.5
15	NW/17	2380	15.8±0.5	4.2	1.4	31.5

The root system was sorted into four classes based on diameter: 0–10 mm (R1), 10–25 mm (R2), 25–100 mm (R3), and 100–150 mm (R4) [22–24]. Therefore, there were a total 294 samples (14 years of decay × 3 sites × 7 components). The lengths and diameters of the above- and below-ground woody debris and the linked roots were measured; the fresh woody debris, bark and linked roots of each stump system were weighed; and all components were collected. Because cut wood surfaces can provide entry points for decomposition-inducing organisms, causing the topmost portion of the stump to decay faster [24, 25], the top 5 cm of each above-ground woody debris sample was removed using a chainsaw and discarded. Then, a 5-cm-thick disc was cut and weighed. For the below-ground woody debris, samples were taken from the upper, middle and lower portions of each below-ground woody debris sample and weighed. All weighed components were taken back to the laboratory.

After the soil was removed, water displacement was used to measure the volume of each component [26]. All samples were subsequently dried in an oven (70°C) for at least 48 h until reaching a constant weight. Bulk density values (*ρ*, in kg∙m^-3^) were defined as the oven-dried mass divided by the green volume [22, 27], calculated using the following formula:

ρ=m/v (1)

where *m* is the dry mass in kg, and *v* is the volume in m^3^. The initial bulk density (*ρ*_*0*_) for each component of the stump system was estimated as an average of 3 corresponding components of stump system sampled similarly in the plantation that was harvested in the year 2013.

We defined the mass remaining as follows:

$$Mass\;loss=(1\;\text{-}\;\rho_{t}/\rho_{0})$$ (2)

where *ρ*_*0*_ is the initial bulk density and *ρ*_*t*_ is the bulk density of the remaining components at time *t* during the studied periods.

To model the decomposition process and determine the annual decomposition rate constants we used a single exponential model [28] for mass loss:

$$Y=a\times e^{\text{-}kt}$$ (3)

where *t* is the time of decomposition, in years; *Y* is the mass remaining at during the studied periods, *k* is the annual decomposition rate constant, in years^-1^, and *a* is the correction factor.

To determine the decomposition parameters for stump systems, the mass of stump systems was estimated as the sum of all components (above-ground woody debris, below-ground woody debris, bark and linked roots with different diameters) calculated by multiplying the volume of wood by bulk density (*ρ*, formula(1)). Then, the annual decomposition rate constants (*k* value) and mass loss were calculated by analogy with calculations of these parameters for other components of stump systems (formula (2)-(3)).

Finally, the collected samples were ground and sifted through a 0.25 mm sieve. Two 1.000 g air-dried subsamples were taken. One subsample was shaken with a mixed solution of 100 ml 0.1 mol·L^-1^ NaOH + 0.1 mol·L^-1^ Na_4_P_2_O_7_·10H_2_O for 10 min and heated in boiling water (i.e., temperature of 100°C) for 30 m in (solution A), and humus carbon was extracted with the same mixed solution in the use of another 1.000 g subsample heated at 80°C for 1 h (solution B) [29–31]. After cooling solutions A and B to room temperature, the black solution, containing dissolved alkali-soluble humic substances, was passed through a 0.45 μm filter to eliminate insoluble granules. Solution A was subsequently analyzed using a UV-visible spectral analyzer (TU-1901, Puxi, Beijing, China). The humic substance concentration of solution B was analyzed using a TOC analyzer (multi N/C 2100, Analytic Jena, Thüringen, Germany). In addition, the initial organic carbon concentration was determined by dichromate oxidation. The degree of humification (HD) was obtained using the following formula:

HD(%)=100×HS/OC (4)

where *HS* is the humic substance concentration and *OC* is the organic carbon concentration.

The UV-visible spectra of humic substances are nearly straight on a logarithmic scale; thus, the slopes of these spectra have been widely used as an index for the degree of humification [32]. The logarithm of absorbance at 400 nm and 600 nm is abbreviated as ΔlogK, and the ratio of absorbance at 465 nm and 665 nm is abbreviated as E4/E6, both of which often appear in the literature [32, 33]. These two variables are obtained by the following equations.

ΔlogK=log(A400/A600) (5)

$$E4/E6=A_{465}/A_{665}$$ (6)

where A400, A465, A600, A665 are the absorbances at 400 nm, 465 nm, 600 nm, and 665 nm, respectively, in 0.1 mol·L^-1^ NaOH.

Moreover, the ΔlogK and E4/E6 values decrease with the development of conjugation structures in the macromolecules of humic substances; thus, a lower ΔlogK or E4/E6 value indicates a higher degree of humification [34, 35].

### Statistical analyses

One-way analysis of variance (ANOVA) was used to test for significant differences (*p* < 0.05 and *p* < 0.01) in the mass loss, E4/E6, ΔlogK values and degree of humification (HD) among the samples of each component of the studied stump systems during the 15-year decay process. Two-way ANOVA was used to test the effects of years of decay, different components and their interactions on the mass loss, E4/E6, ΔlogK values and degree of humification of the stump systems. The relationship between years of decay and each component of the stumps was determined using Pearson’s correlation coefficient.

## Results

### Mass loss

**Fig 1:**
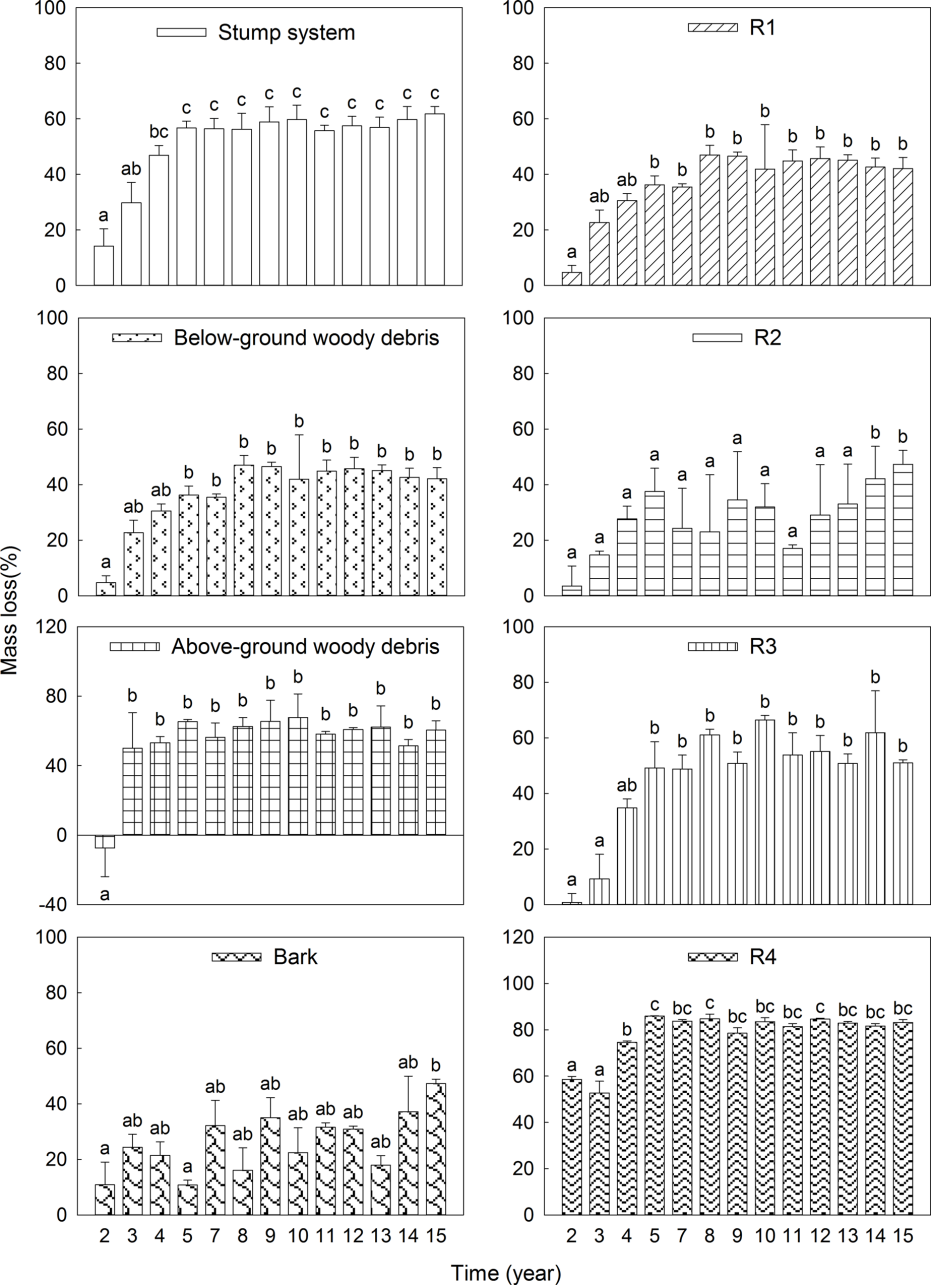
Mass loss of stump systems with humification process in the *P*. *massoniana* plantations (mean ± *SE*, *n* = 3). Different lowercase letters indicate significant differences among different durations of decay (*p* < 0.05). R1, 0 mm < diameter ≤ 10 mm; R2, 10 mm < diameter ≤ 25 mm; R3, 25 mm < diameter ≤ 100 mm; R4, diameter > 100 mm.

Mass loss for stump system showed a significant negative relationship with the years of decay (Table 2), implying mass loss over time (Fig 1). Mass losses for the studied 15 years were 61.8% for the whole stump system, 60.4% for above-ground woody debris, 42.1% for below-ground woody debris, 47.3% for bark, 69.9% for R1, 47.3% for R2, 51.0% for R3, and 83.2% for R4. The annual decomposition rate constants (*k* values) for below-ground woody debris, above-ground woody debris and bark were 0.03, 0.04, and 0.03, respectively; above-ground woody debris had the largest *k*. For the studied root systems, the annual decomposition rate constants for R1, R2, R3, and R4 were 0.09, 0.03, 0.06, and 0.06, respectively; R1 had the largest *k* value and R3 and R4 the next largest (Table 3).

**Table 2:** Pearson’s correlations between mass loss, degree of humification (HD), E4/E6 and ΔlogK of different stump components during 15 years of decay.

	Years of decay	HD	E4/E6	ΔlogK	Mass loss
Years of decay	1				
HD	0.14*	1			
E4/E6	-0.27**	0.06	1		
**Δ**logK	-0.20	-0.17*	0.01	1	
Mass loss	0.59**	0.22*	-0.04	-0.10	1

* Significant at the 0.05 probability level.

** Significant at the 0.01 probability level.

**Table 3:** Parameters of the single-exponential decomposition model for stump systems.

Components	Regression equation	R^2^	*k*	T_0.5_	T_0.95_
Stump system	y = 0.7 e^-0.05t^	0.6	0.05	5.6	54.6
Below-ground woody debris	y = 0.8 e^-0.03 t^	0.5	0.03	13.2	82.6
Above-ground woody debris	y = 0.6 e^-0.04 t^	0.2	0.04	3.2	64.4
Bark	y = 0.9 e^-0.03 t^	0.5	0.03	21.7	111.0
R1	y = 0.9 e^-0.09 t^	0.9	0.09	6.6	32.7
R2	y = 0.9 e^-0.03 t^	0.5	0.03	18.7	96.0
R3	y = 0.8 e^-0.06 t^	0.5	0.06	7.5	48.0
R4	y = 0.3 e^-0.06 t^	0.4	0.06	7.9	29.9

R1, 0 mm < diameter ≤ 10 mm; R2, 10 mm < diameter ≤ 25 mm; R3, 25 mm < diameter ≤ 100 mm; R4, diameter > 100 mm.

### E4/E6

E4/E6 is the abbreviation for the ratio of absorbance at 465 and 665 nm, which is an index of the degree of humification. E4/E6 values of the stump system eventually decreased (Table 4), showing a very significant negative relationship with years of decay (Table 2). The years of decay (F = 28.44, *p* < 0.01; Table 5), different components (F = 47.49, *p* < 0.01; Table 5) and their interactions (F = 7.95, *p* < 0.01; Table 5) significantly influenced the E4/E6 values. Different components showed almost the same trend as the stump system. Overall, the variation trend fluctuated. E4/E6 values for coarse woody debris and different root systems initially decreased during the first 3–4 years then increased until 8–9 years of decay, finally showing a decreasing trend.

**Table 4:** Dynamics of E4/E6 in the humification process of stump systems from the studied *P*. *massoniana* stumps (mean ± *SE*, *n* = 3). Different lowercase letters indicate significant differences among different durations of decay (*p* < 0.05).

Years of decay	Stump system	Below-ground woody debris	Above-ground woody debris	Bark	R1	R2	R3	R4
1	12.7(0.2)^a^	12.7(0.2)^ab^	11.7(0.3)^ab^	13.6(0.2)^ab^	12.3(0.1)^ab^	12.0(0.0)^ab^	11.5(0.1)^ab^	15.0(0.7)^a^
2	12.8(0.3)^a^	13.3(0.0)^ab^	11.1(0.3)^abc^	12.9(0.5)^ab^	12.7(0.2)^a^	12.5(0.5)^a^	13.0(0.4)^a^	14.2(0.9)^ab^
3	10.8(0.3)^efg^	11.1(0.6)^bcd^	5.2(0.6)^e^	13.5(0.6)^ab^	11.8(0.2)^bcd^	12.9(0.3)^a^	10.6(0.1)^ab^	10.3(0.8)^de^
4	11.1(0.2)^cdef^	9.1(0.3)^d^	12.2(0.5)^ab^	9.6(0.9)^c^	10.7(0.2)^f^	12.8(0.4)^a^	11.2(0.7)^ab^	12.2(0.2)^bcd^
5	12.1(0.2)^abc^	13.2(0.5)^ab^	11.1(0.5)^abc^	12.9(0.5)^ab^	12.1(0.1)^abc^	12.4(0.1)^a^	11.6(1.0)^ab^	11.8(0.1)^bcde^
7	11.9(0.2)^abcde^	12.3(0.4)^abc^	11.8(0.5)^ab^	12.5(0.1)^ab^	11.0(0.3)^ef^	11.6(0.4)^ab^	11.6(0.4)^ab^	12.2(0.2)^bcd^
8	12.9(0.1)^a^	11.8(0.4)^bcd^	11.6(0.3)^ab^	14.3(0.4)^a^	12.0(0.2)^bc^	12.8(0.2)^a^	13.2(0.3)^a^	14.3(0.4)^ab^
9	12.2(0.2)^abc^	13.6(1.0)^ab^	10.3(0.5)^abc^	13.3(0.2)^ab^	11.2(0.0)^def^	11.9(0.2)^ab^	12.0(0.2)^a^	13.2(0.4)^abc^
10	12.4(0.3)^ab^	15.1(0.6)^a^	10.6(0.1)^abc^	13.6(0.8)^ab^	12.0(0.0)^bc^	12.0(0.8)^ab^	9.2(0.4)^bc^	14.0(0.7)^ab^
11	12.0(0.2)^abcd^	11.9(0.5)^bcd^	12.5(0.2)^a^	13.1(0.3)^ab^	11.2(0.0)^def^	11.7(0.2)^a^	11.3(0.5)^ab^	12.5(0.4)^abcd^
12	12.2(0.1)^bcdef^	11.3(0.0)^bcd^	10.8(0.9)^abc^	13.6(0.6)^ab^	11.6(0.0)^cde^	13.0(0.0)^ab^	6.7(0.5)^c^	11.7(0.5)^bcde^
13	11.0(0.1)^defg^	9.2(0.8)^d^	8.9(0.4)^cd^	11.7(0.0)^bc^	11.6(0.0)^cde^	11.8(0.1)^cd^	11.8(0.1)^ab^	11.6(0.3)b^cde^
14	10.6(0.2)^fg^	9.7(0.3)^cd^	7.9(0.4)^d^	12.8(0.1)^ab^	11.7(0.0)^bcde^	10.5(0.1)^b^	10.9(1.1)^ab^	11.1(0.7)^cde^
15	9.9(0.4)^a^	9.7(1.0)^cd^	9.9(0.6)^bcd^	11.9(0.2)^abc^	10.8(0.0)^f^	10.5(0.5)^b^	7.7(0.1)^c^	9.1(0.2)^e^

R1, 0 mm < diameter ≤ 10 mm; R2, 10 mm < diameter ≤ 25 mm; R3, 25 mm < diameter ≤ 100 mm; R4, diameter > 100 mm.

**Table 5:** Two-way ANOVA of the mass loss, degree of humification (HD), E4/E6 and ΔlogK related to the different years of decay and different components of the studied stump systems.

	df	F			
		HD	Mass loss	E4/E6	ΔlogK
Years of decay	13	29.90**	35.48**	28.44**	3.94**
Components	6	27.74**	62.67**	47.49**	21.24**
Years of decay (Components)	78	6.53**	1.75**	7.95**	2.31**

** Significant at the 0.01 probability level.

### ΔlogK

ΔlogK values (the slopes of UV-visible spectra of ratio of absorbance at 400 nm and 600 nm on a logarithmic scale, which is an index of the degree of humification) of the stump system decreased, with lower ΔlogK values found during the later phase (Table 6) and a very significant negative relationship between ΔlogK values and years of decay (Table 2). The years of decay (F = 3.94, *p* < 0.01; Table 5), different components (F = 21.24, *p* < 0.01; Table 5) and their interactions (F = 2.31, *p* < 0.01; Table 5) significantly influenced the ΔlogK values. Overall, various trends of the different components were similar (except for R1, R2), and the ΔlogK values of coarse woody debris and different root systems showed a decreasing trend during the studied period.

**Table 6:** Dynamics of ΔlogK in the humification process of stump systems from the studied *P*. *massoniana* stumps (mean ± *SE*, *n* = 3). Different lowercase letters indicate significant differences among different durations of decay (*p* < 0.05).

Years of decay	Stump system	Below-ground woody debris	Above-ground woody debris	Bark	R1	R2	R3	R4
1	0.9(0.0)^a^	0.9(0.0)^ab^	1.1(0.2)^abc^	0.9(0.0)^ab^	0.9(0.0)^abc^	0.8(0.0)^abc^	1.0(0.0)^a^	1.0(0.0)^a^
2	1.0(0.0)^a^	1.3(0.0)^b^	1.3(0.1)^a^	0.8(0.0)^b^	0.8(0.0)^cd^	0.8(0.0)^abc^	0.8(0.0)^a^	0.9(0.0)^a^
3	1.0(0.0)^a^	1.1(0.2)^ab^	1.2(0.1)^ab^	0.9(0.0)^ab^	0.9(0.0)^bcd^	0.9(0.0)^abc^	0.9(0.1)^a^	0.9(0.0)^a^
4	1.0(0.0)^a^	1.1(0.1)^ab^	0.9(0.0)^bc^	0.9(0.1)^a^	1.0(0.1)^a^	1.0(0.0)^a^	1.0(0.1)^a^	0.9(0.0)^a^
5	0.9(0.1)^a^	1.0(0.1)^ab^	1.0(0.0)^bc^	0.8(0.0)^b^	0.8(0.0)^d^	0.8(0.0)^bc^	1.1(0.3)^a^	0.9(0.0)^a^
7	0.9(0.0)^a^	0.9(0.1)^ab^	1.0(0.0)^abc^	0.8(0.0)^ab^	0.8(0.0)^cd^	0.9(0.1)^abc^	0.9(0.1)^a^	0.9(0.1)^a^
8	1.0(0.0)^a^	1.1(0.0)^ab^	1.0(0.0)^abc^	0.9(0.0)^ab^	0.8(0.0)^bcd^	0.9(0.0)^abc^	0.9(0.0)^a^	1.0(0.1)^a^
9	0.9(0.0)^a^	1.0(0.1)^ab^	0.9(0.0)^bc^	0.8(0.0)^ab^	0.8(0.0)^bcd^	0.8(0.0)^abc^	0.9(0.0)^a^	1.0(0.1)^a^
10	1.0(0.0)^a^	1.1(0.0)^ab^	0.9(0.0)^bc^	0.8(0.0)^ab^	1.0(0.0)^a^	1.0(0.1)^ab^	1.0(0.0)^a^	1.0(0.0)^a^
11	0.9(0.0)^a^	1.0(0.0)^ab^	1.0(0.0)^ab^c	0.9(0.0)^ab^	0.8(0.0)^bcd^	0.8(0.0)^abc^	0.9(0.1)^a^	1.0(0.0)^a^
12	0.8(0.0)^b^	0.8(0.1)^a^	0.9(0.0)^bc^	0.8(0.0)^ab^	0.8(0.0)^bcd^	0.9(0.0)^abc^	0.7(0.0)^a^	1.0(0.0)^a^
13	0.9(0.0)^a^	1.3(0.2)^b^	0.9(0.0)^bc^	0.8(0.0)^ab^	0.8(0.0)^cd^	0.8(0.1)^bc^	0.9(0.0)^a^	1.0(0.0)^a^
14	0.9(0.0)^b^	0.9(0.1)^ab^	0.8(0.0)^c^	0.9(0.0)^ab^	0.9(0.0)^ab^	0.7(0.0)^c^	0.9(0.1)^a^	1.0(0.1)^a^
15	0.9(0.0)^b^	0.9(0.0)^ab^	0.9(0.0)^bc^	0.8(0.0)^b^	0.9(0.0)^abc^	0.9(0.0)^abc^	0.7(0.0)^a^	1.0(0.0)^a^

R1, 0 mm < diameter ≤ 10 mm; R2, 10 mm < diameter ≤ 25 mm; R3, 25 mm < diameter ≤ 100 mm; R4, diameter > 100 mm.

### Degree of humification

**Fig 2:**
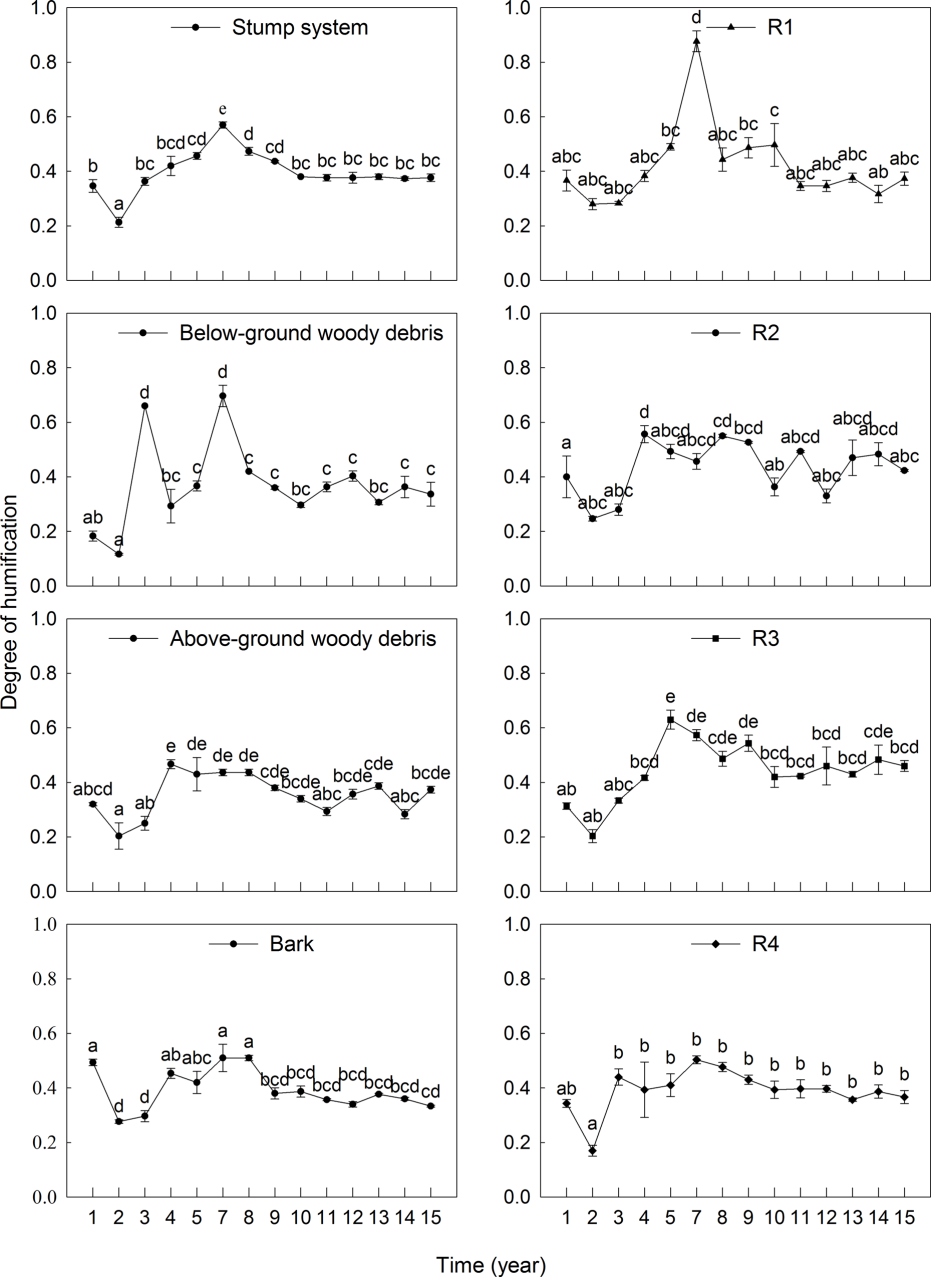
Dynamics of the degree of humification in the humification process of stump systems from the studied *P*. *massoniana* plantation (mean ± *SE*, *n* = 3). Different lowercase letters indicate significant differences among different durations of decay (*p* < 0.05). R1, 0 mm < diameter ≤ 10 mm; R2, 10 mm < diameter ≤ 25 mm; R3, 25 mm < diameter ≤ 100 mm; R4, diameter> 100 mm.

Years of decay (F = 29.90, *p* < 0.01; Table 5), different components (F = 27.74, *p* < 0.01; Table 5) and their interactions (F = 6.53, *p* < 0.01; Table 5) significantly influenced the degree of humification. There was a significant positive relationship between the degree of humification and the years of decay (Table 2). The dynamics of the degree of humification in the stump system were fluctuated. The maximum degree of humification was observed after seven years of decay, after which the degree of humification decreased; however, the final value for the degree of humification was higher than that at the initial time. After 15 years of decay, the detected degree of humification of stump systems was 0.3–0.5. Various components showed different trends, but they generally showed fluctuation (Fig 2). Over the 15-year decay process, the observed degree of humification was 0.1–0.7 for the below-ground woody debris, 0.2–0.5 for the above-ground woody debris, and 0.3–0.5 for bark. For the studied root systems, the degree of humification was 0.3–0.4 for R1, 0.3–0.6 for R2, 0.2–0.5 for R3, and 0.3–0.5 for R4 (Fig 2). All of these values, except that for bark, were increased compared to the initial degree of humification (Fig 2). The below-ground woody debris exhibited a larger degree of humification than bark and above-ground woody debris; R2, R3 and R4 showed larger degrees of humification than R1 based on the studied root systems. Therefore, the below-ground woody debris and roots with large diameters (R2, R3 and R4) had higher degrees of humification than other parts.

## Discussion

### Mass loss and humification

The decay of CWD is complex and controlled by many factors (such as the substrate quality, composition and environment) [24, 36]. Generally, decay processes include mass loss and humification. Stump systems are vital components of both the structure and function of forests; stumps can provide habitants for animals [37] and microbes [38], serve as water reservoirs [39], and provide carbon sequestration, among other roles. The mass loss process is a way for nutrients to return to the soil, and the humification process is a way to preserve nutrients. The structural components of stump systems directly impact the decomposition patterns. Based on our analysis, mass loss had a significantly positive relationship with the degree of humification (Table 2), and the degree of humification accounted for a vital portion of the mass loss (nearly 50%), which means that a large proportion of the decomposed organic materials was fixed in the form of humus. These results are partly consistent with studies that have documented a relatively high degree of litter humification [36, 40].

Lower E4/E6 and ΔlogK values indicate a higher degree of humification, and the ΔlogK and E4/E6 values of stump systems showed a negative relationship with the degree of humification (Table 2). Simple materials accumulated in the stump systems may hinder the development of conjugation structures in the macromolecules of humic substances, thus resulting in increased E4/E6 and ΔlogK values. Moreover, humus accumulation is primarily driven by microbial polymerization [41]; populations of microorganisms and soil fauna require time to grow, which will affect the humification [42–44] of stump systems. In addition, previously formed humus may be mineralized at the same time [45], and humus with different molecular sizes might exist in harmony or transmute into each other, which is consistent with Kononova [46]. Consequently, in our study, the degree of humification fluctuated during the early stages of stump decay. Later in the decomposition process, simple organic materials that microorganisms could use decreased in quantity. Humus contains hard-to-degrade substances, such as cellulose and phenol, were the complexes of polymers of humus [47]. As these humic substances accumulated during the degradation process, the E4/E6 and ΔlogK values decreased, indicating an increasing degree of humification.

### Coarse woody debris

The decay processes of the studied above- and below-ground woody debris and bark varied. For coarse woody debris, we found that annual decomposition rate constants (*k* values) for above-ground woody debris were larger than those for below-ground woody debris and that bark had the lowest *k* values (Table 3). Compared to previous studies, our *k* values for *P*. *massoniana* stump systems during 15 years of decay (0.05 year^-1^) were larger than those for *Picea abies* (0.04 year^-1^) and *P*. *sylvestris* (0.04 year^-1^) but smaller than those for *P*. *radiata* (0.1 year^-1^) and *Betula pubescens* (0.07 year^-1^) [14, 48]. Further, the *k* value for above-ground woody debris (0.04 year^-1^) was lower than that of *P*. *radiata* (0.07–0.14 year^-1^) [14, 49, 50]; *k* values for bark (0.03, year^-1^) were smaller than for *P*. *sylvestris* (0.03 year^-1^) and *Betula pubescens* (0.06 year^-1^) but larger than for *Picea abies* (0.01 year^-1^) [48]. However, the degree of humification of above-ground woody debris was smaller than those for bark and below-ground woody debris. The decomposition rate of CWD is known to be a function of comprehensive factors (such as vegetation zone, site conditions, tree species, size and so on). Differences in the degrees of humification among these types of CWD might be associated with the influence of ambient environmental factors (e.g., rainfall and freeze-thaw) [36], CWD qualities [51] and organisms.

Above- and below-ground woody debris might have similar chemical compositions. Compared with below-ground woody debris, above-ground woody debris is subject to more environmental disturbances (such as solar radiation, precipitation and predators activities), which would facilitate colonization by wood-boring insects and microorganisms, accelerating physical fragmentation and degradation into its chemical constituents [52, 53], so relatively large mass loss was detected in above-ground woody debris (Fig 1). Therefore, there was less accumulation of recalcitrant materials, such as lignin and phenols, which were identified as the polymers of humic substances [47], than in below-ground woody debris and bark. In contrast, humification largely depended on the composition of fauna and microorganisms [41]. To certain extent, more favorable ambient temperature and moisture conditions may promote the growth and activity of soil microorganisms [54], together with smaller *k* values (Table 3), and the humus accumulation accelerated. In conclusion, below-ground woody debris had a higher degree of humification (Fig 2). Although bark seemed to be more vulnerable to the invasion of environment factors and decomposers for act as protector for stump wood [55, 56], studies have demonstrated that bark is more anti-corrosive than other tree components [57, 58], and our results (Table 3) are consistent with previous studies showing that bark had lower *k* values than wood [49, 59]. Higher levels of recalcitrant materials might facilitate the humification process of bark; consistently, lower *k* values and higher degrees of humification than above-ground woody debris of bark were identified in our study (Table 3, Fig 2).

### Root system

Roots with different diameters showed varying dynamics, implying diverse decay trends in the root system. It is generally accepted that fine roots decompose more quickly than coarse roots [60, 61], mainly because fine roots have a larger surface-to-volume ratio, which often leads to increasing fragmentation and decomposition rates [20]. In our study, roots with the smallest diameter (R1) had the largest *k* values, but among the other roots (R2, R3, R4), the *k* values increased with increasing root diameter, implying that root decomposition and root size in our study were not consistent. Such unclear relationships between root size and decomposition rate have also been found in previous studies. An experiment on four dominant species (*Acer saccharum*, *Betula alleghaniensis*, *Fagus grandifolia*, *Picea rubens*) in a northern hardwood ecosystem found that the decomposition rate of coarse roots was nearly 10 times greater than that of fine roots early in the decay process [62]. In contrast, Chen et al found that root diameter (10–50 mm vs. 50–150 mm) did not significantly influence the decomposition rate, which is probably attributable to the similarities in root structural components [22]. A study using the Kaplan-Meier method in survival analysis for *Fraxinus mandshurica* and *Larix gmelinii* found that decay of fine roots was faster than that of coarse roots [63]. In the *P*. *massoniana* root system, the factors influencing decomposition seemed to be more complex.

Fine roots (diameter < 10 mm) clearly showed more rapid decay than coarse root (diameter > 10 mm). However, when considering only roots with diameters of 10–25 mm, 25–100 mm and >100 mm, the decomposition rate in our study increased with root diameter. This is likely due to secondary defense substances, such as tannins in the cell walls of root bark, which make root difficult to decompose. Bark tissue is present at higher proportions in the cross sectional areas of fine root, which may also result in slow degradation. Moreover, small roots have higher N concentrations, which make the formation of nitrogen-lignin compounds easier [22, 64]; therefore, the decomposition of small root may be restrained. This result regarding root system decomposition in our study might also be attributable to the classification of root systems, considering that high variability in the diameter of woody roots within each size class may preclude the detection of an effect of size on root decomposition. As for the degree of humification of the root system, it was more likely controlled by root qualities, given that roots with larger *k* values showed lower degrees of humification (Table3, Fig 2).

### Coarse woody debris and root systems

Many studies have presented diverse viewpoints on the relationship between root system and coarse woody debris varying with position, species, size and age. Our study indicated that there were different degrees of mass loss and humification in coarse woody debris and that humification could be attributed mainly to differences in the proportions of chemical substances and in physical characters [3]. Palviainen and Marjo documented that roots had lower *k* values (0.02–0.03 year^-1^) than CWD (0.04 year^-1^) [19]; Garrett et al indicated that coarse roots decomposed faster (*k* = 0.2 year^-1^) than stems and logs (*k* = 0.1 year^-1^) [14]. In our study, the root system decomposed more quickly than above-ground woody debris, below-ground woody debris and bark (Table 3), although the degree of humification of the root system (0.2–0.6) was lower than that of below-ground woody debris (0.1–0.7) but somewhat larger than those of above-ground woody debris (0.2–0.5) and bark (0.2–0.5) (Fig 2). Therefore, the contribution of *P*. *massoniana* root systems to soil organic matter might equal or exceed that of coarse woody debris. This result is partly in agreement with previous studies reporting that soil organic matter and nutrient pool turnover by the roots can equal or exceed CWD inputs [65, 66].

## Conclusions

All components of the studied stump systems exhibited relatively high degrees of humification. By comparing the stump system degradation (mass loss) and humification (the formation of organic matter), we found that more than 50% of the decomposed organic material was fixed in the form of humic substances. The degree of humification increased with years of decay during the studied period. This result suggests that the below-ground woody debris and the root system played more important roles in humification, carbon sequestration and soil fertility maintenance than the other components of the stump system. The study of the decay process of managed Masson pine stump systems in North China has implications for the management of soil strength, biodiversity and nutrient dynamics. With the increasing soil erosion of managed forests, our data may assist in predicting soil erosion in these ecosystems. The results of this study will help to provide a scientific basis for the sustainable operation and management of Masson pine plantations.

## Supporting Information

S1 File Original date of the study. (Mass loss, bulk density, degree of humification, E4/E6, ΔlogK).
(XLSX)
